# Aerosol reduction efficacy of different intra-oral suction devices during ultrasonic scaling and high-speed handpiece use

**DOI:** 10.1186/s12903-022-02386-w

**Published:** 2022-09-06

**Authors:** Krystyna Piela, Paddy Watson, Reuben Donnelly, Marilyn Goulding, Fiona L. Henriquez, William MacKay, Shauna Culshaw

**Affiliations:** 1 Oral Sciences, Glasgow Dental Hospital and School, College of Medical, Veterinary and Life Sciences, University of Glasgow, Glasgow, G2 3JZ UK; 2 Global Clinical Affairs, Dentsply Sirona, York, PA USA; 3 School of Health and Life Sciences, University of the West of Scotland, Lanarkshire Campus, Blantyre, G72 0HL UK

**Keywords:** Aerosol, COVID-19, Dental clinics, Dental equipment, Dental hygienists, Dental office, Dental scaling, SARS-Cov-2, Suction

## Abstract

**Background:**

The COVID-19 pandemic led to significant changes in the provision of dental services, aimed at reducing the spread of respiratory pathogens through restrictions on aerosol generating procedures (AGPs). Evaluating the risk that AGPs pose in terms of SARS-CoV-2 transmission is complex, and measuring dental aerosols is challenging. To date, few studies focus on intra-oral suction. This study sought to assess the effectiveness of commonly used intra-oral suction devices on aerosol mitigation.

**Methods:**

Ultrasonic scaling and high-speed handpiece procedures were undertaken to generate aerosol particles. Multiple particle sensors were positioned near the oral cavity. Sensor data were extracted using single board computers with custom in-house Bash code. Different high-volume and low-volume suction devices, both static and dynamic, were evaluated for their efficacy in preventing particle escape during procedures.

**Results:**

In all AGPs the use of any suction device tested resulted in a significant reduction in particle counts compared with no suction. Low-volume and static suction devices showed spikes in particle count demonstrating moments where particles were able to escape from the oral cavity. High-volume dynamic suction devices, however, consistently reduced the particle count to background levels, appearing to eliminate particle escape.

**Conclusions:**

Dynamic high-volume suction devices that follow the path of the aerosol generating device effectively eliminate aerosol particles escaping from the oral cavity, in contrast to static devices which allow periodic escape of aerosol particles. Measuring the risk of SARS-CoV-2 transmission in a dental setting is multi-factorial; however, these data suggest that the appropriate choice of suction equipment may further reduce the risk from AGPs.

**Supplementary Information:**

The online version contains supplementary material available at 10.1186/s12903-022-02386-w.

## Background

The importance of minimising airborne pathogens in dentistry has been brought sharply into focus by the coronavirus disease 2019 (COVID-19) pandemic. Severe acute respiratory syndrome coronavirus 2 (SARS-CoV-2) and other respiratory pathogens are spread via respiratory droplets and aerosols [1]; these respiratory secretions may be generated by coughing and breathing, but there is also concern that they may be spread via dental instrument aerosols.

The COVID-19 pandemic led to restrictions being imposed on the provision of dental services, many of which aimed to minimise the generation of dental aerosols and exposure to respiratory secretions, therefore potentially reducing the risk of SARS-CoV-2 transmission in the dental setting. This impacted the number of patients that dental care professionals could see and the treatments that could be provided, ultimately compromising patient access to dental care.

There is ambiguity in what constitutes an aerosol generating procedure (AGP); a lack of evidence on the amount of aerosol that poses a risk of transmission, and a lack of evidence around supposed risk mitigation measures. According to ‘A Review of Aerosol Generation Mitigation in International Dental Guidance’, [2] many country-specific recommendations did not provide a specific definition of an AGP and guidelines differ between regions. Among the recommendations there is generally a consensus that high-speed handpieces are a source of aerosol. In the absence of evidence, perception of other instruments and procedures varies from country to country. For example, Brazil and Mexico exclude powered scalers from their list of AGP instruments; whereas numerous countries, including the USA and UK, state that the use of all types of dental handpieces, powered scalers and 3-in-1 syringes should be treated as AGPs [2]. Dentists surveyed during the pandemic expressed confusion regarding the current guidance in-place, particularly in terms of what was appropriate practice to effectively mitigate AGPs, and ultimately concerns were raised regarding the impact this ongoing uncertainty may have on service provision and patient care [3]. Furthermore, despite the general shift towards remobilisation of medical and dental services after the pandemic peak, dentists in some countries are still restricted in the number of AGPs they can realistically provide due to the existing guidance regarding fallow-time [4].

There are a wide variety of methods to measure aerosols in a dental setting; these have included culturing of settle plates [5–7], fluorescent-irrigant settling onto filter paper discs [8–12], bacteriophage dispersal [13], and aerosol particle measurements [14–19]. To date, there is no ‘industry standard’ measurement of aerosol or droplet dispersion. In the absence of a ‘gold standard’ for measurements, and the absence of an ‘acceptable threshold’ of aerosol, measuring aerosol mitigation remains challenging. Many studies have evaluated aerosol mitigation using extra-oral high-volume suction devices, [7, 10, 15, 16, 19]. In some settings, these devices show effective aerosol removal but these devices are not widely used. A recent study demonstrated that a combination of intra-oral high and low-volume suction could reduce aerosols generated from both the ultrasonic scaler and the high-speed handpiece to background levels immediately following completion of AGPs, however, they concluded that the aerosol exposure cannot be fully eliminated [17]. Furthermore, a recent study attempted to evaluate the level of aerosols generated by dental procedures to attempt to quantify these differences. They showed that high-speed handpieces generated at least ten times more aerosol particles than ultrasonic scaling and demonstrated that high-volume intra-oral suction was effective at mitigating aerosol generated from ultrasonic scaling to background levels [18].

High or low-volume intra-oral suction devices are widely used by dental professionals as their primary form of suction, however, limited evidence is available for their effectiveness on aerosol mitigation. The aim of this study was to evaluate a variety of commonly used intra-oral suction devices and measure their effects on aerosol mitigation, in real-time, using particle sensors during ultrasonic scaling and high-speed handpiece AGPs.

## Methods

### Study setting

The study setup within the University of Glasgow Dental Clinical Research Facility consisted of a dental mannikin head attached to a dental unit, complete with standard built-in high and low-volume suction, coolant and air-turbine functions (Additional file 1), with a separate portable ultrasonic scaling unit. As part of the hospital ventilation assessment the room was assessed as zero air changes per hour and during procedures the windows were kept closed with no mechanical ventilation.

### Particulate sensors positioning and data acquisition

Two Plantower PMS5003 particulate matter sensors (Plantower, China) [20] were used for the duration of the study. These sensors utilise laser scattering to determine particle sizes and detect particle sizes within the aerosol and droplet ranges, from 0.3 to 10 µm in diameter [21], with readings provided for every 0.1 L of air intake. The sensors were used in two different arrangements. Pilot experiments identified the optimum position for sensor placement, to both maximise detection of particles escaping from the oral cavity but also minimise restriction of access for both operator and assistant. The first of these arrangements, termed the ‘single sensor’ setup, involved a single sensor placed 10 cm vertically above the upper and lower central incisors with a funnel attachment to help maximise particle capture. The second arrangement, termed the ‘dual sensor’ setup, involved two sensors being placed in the nose and chin region, 5 cm from the upper and lower incisal edges, with readings taken simultaneously (Additional file 1). The sensors were connected to single board computers (Raspberry Pi, UK). Using a bespoke in-house Bash code (Additional file 2) the particle readings were assimilated into fixed width text files that were then analysed with both Microsoft Excel and GraphPad Prism.

### Aerosol-generating devices

Ultrasonic scaling was carried out using a Cavitron Touch® Scaling System (Dentsply Sirona, US) connected to a Cavitron® DualSelect™ Dispensing System (Dentsply Sirona, US), with a Cavitron® Powerline® 1000 30 K Ultrasonic Insert (Dentsply Sirona US) (Additional file 3A). 0.89% saline was used as a coolant for all ultrasonic procedures, measured at a flowrate of 18.0 ml/min. The ultrasonic power was set to 100% for all procedures. High-speed turbine/handpiece treatment was carried out using a Midwest Stylus® Plus Handpiece (Dentsply Sirona, US) (Additional file 3B) with a flat end cylindrical diamond bur (ISO: 111-012 M (837)). 0.89% saline was used as a coolant for all high-speed handpiece procedures, measured at a flowrate of 68.0 ml/min.

### Suction devices

A total of six dental unit suction devices were used for the ultrasonic scaling arm of the study. These were divided into ‘dynamic’ suction devices which closely follow the aerosol generating instrument throughout the procedure: Standard high-volume suction (referred to as ‘standard HVE’) (Additional file 3C), Purevac® HVE System [22] which included the lightweight hose and adapter (referred to as ‘Purevac (+H)’; Dentsply Sirona, US) and Purevac® HVE Mirror Tip connected directly to the suction port (referred to as ‘Purevac (−H)’; Dentsply Sirona, US) (Additional file 3D); and ‘static’ suction devices which are hands-free and maintained in the same position throughout the procedure: DryShield® Isolation System [23] (referred to as ‘DryShield’; DryShield, US) (Additional file 3E), Ivory® ReLeaf™ hands-free suction device [24] (referred to as ‘ReLeaf’; Kulzer, US) (Additional file 3F) and standard low-volume suction placed lingual to the lower central incisors (referred to as ‘standard LVE’) (Additional file 3G). For the high-speed handpiece arm of the study, only the standard HVE, Purevac (±H) and DryShield devices were used. In both arms of the study the efficacy of the devices was also compared against a negative control—the absence of suction (no suction) (Additional file 4).

### Ultrasonic scaling protocol

**Fig. 1:**
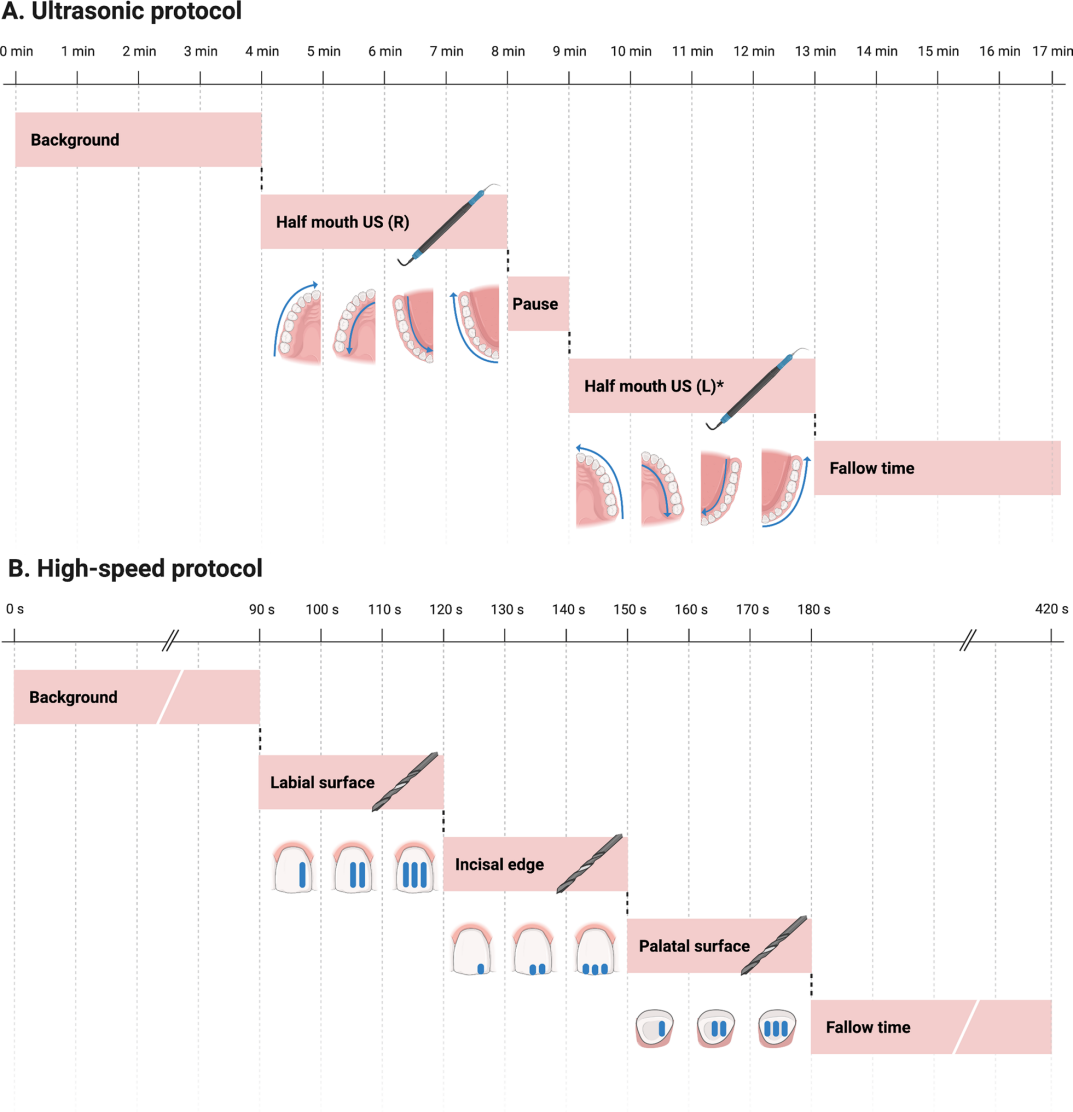
Aerosol generating procedure protocols. Schematics showing the positioning and timing for **A,** Ultrasonic scaling and **B,** High-speed handpiece anterior crown preparation (*Created with BioRender.com*)

As is common practise in dental hygienist treatment, both the ultrasonic scaling and the use of intra-oral suction were operated ‘solo’ by a single operator (PW). For the single sensor setup, the sensor was started and four minutes of background particle levels were recorded to give an initial baseline reading. The sensor was then restarted, and a half-mouth ultrasonic scale of the right-hand side was undertaken for four minutes (Fig. 1A)—this consisted of a predetermined consistent pattern of supragingival scaling, starting in the upper right quadrant at the upper right third molar and then moving in a mesial direction to finish at the upper right central incisor over a one minute period; whilst ensuring the active tip of the ultrasonic scaler was always kept parallel to the long axis of the tooth at the level of the gingival margin, with an equal amount of time spent scaling each tooth to ensure consistency. This was then repeated in the upper right quadrant palatally from the upper central incisor moving distally to the upper third molar. Finally, the same procedure was continued in the lower right quadrant lingually from the lower right third molar to the lower right central incisor, then buccally from the lower right central incisor to the lower right third molar. The sensor was then stopped and the reading saved. Following the half-mouth scale there was a four-minute period of fallow-time. This procedure was completed in the presence of each suction device, used as per the manufacturers’ instructions for use manual [22–24], followed by no suction, for a total of five replicates per device. For the single sensor setup only the right-hand side of the mouth was instrumented as it was not possible to effectively access the left-hand side of the mouth with the ultrasonic scaler, suction device and the funnel in-situ.

In the dual sensor setup, four minutes of background particle levels were recorded immediately prior to an eight-minute full-mouth scale (Fig. 1A)—this consisted of supragingival scaling as per the single sensor setup, with the identical procedure also carried out on the left-hand side of the mouth. A one-minute pause between the right and left sides was included to allow transfer of equipment. At the conclusion of the full-mouth scale, the sensor was stopped and the reading saved, followed by a four-minute period of fallow time. This procedure was completed in the presence of each suction device, used as per the manufacturers’ instructions for use manual, [22–24] followed by no suction, for a total of five replicates per suction device.

### High-speed handpiece protocol

As is common practise in restorative dentistry, the high-speed handpiece procedures were carried out by both an operator (KP) and an assistant (RD). For both the single sensor and the dual sensor setup, the sensor was started and ninety seconds of background particle levels were recorded to given an initial baseline reading. Immediately following this was the use of the high-speed handpiece on the upper right central incisor (Fig. 1B)—this consisted of three depth cuts being made on the labial surface in a mesial to distal direction across a thirty-second period, ten seconds per depth cut, followed by the same procedure on both the incisal and palatal surfaces. This was to mimic the types of cuts used as part of an anterior crown preparation, requiring use of the handpiece in three distinct orientations. At the end of the 90 s the sensor was stopped and the reading saved. After each procedure there was a four-minute period of fallow-time. This procedure was completed in the presence of each suction device, used as per the manufacturers’ instructions for use manual [22, 23], followed by no suction, for a total of five replicates per suction device.

### Data analysis and statistics

For each particle sensor, an output reading of all particles detected between 0.3 and 10 µm was provided for every 0.1 L of air intake. Once each saved file had been processed (Additional file 2), the area under the curve (AUC) for the duration of the AGP was calculated which allowed an evaluation of the variation in particle count as a function of time. Background particle levels showed variations that appeared independent of both temperature and humidity readings. Therefore, to accommodate changeable atmospheric particle levels between experiments, the AUC for the initial background reading was deducted from the AUC for the AGP reading to give a normalised AUC value for analysis, hereby referred to as ‘normalised particle count’.

Statistical analysis was carried out from the mean normalised particle counts ± standard deviation for each device (n = 5) using one-way analysis of variance (ANOVA) with Tukey’s post-hoc test. All data analysis and statistical tests were conducted using MS Excel and GraphPad Prism with a significance level set at 0.05.

## Results

### High-speed handpieces generate greater particle numbers compared with ultrasonic scaling

**Fig. 2:**
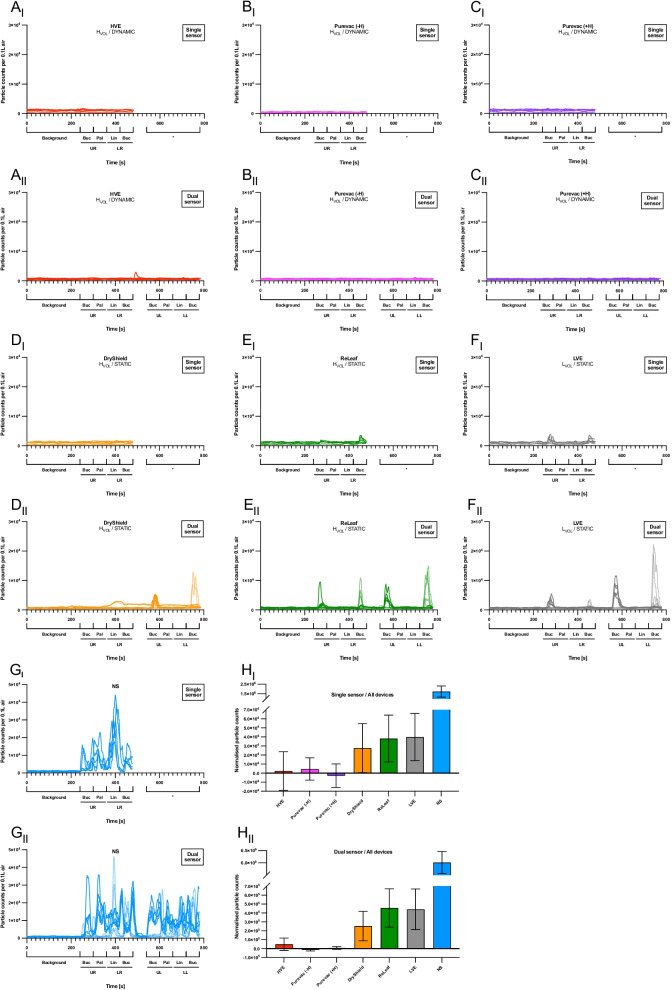
Particle counts for ultrasonic procedures. **A-G**, Line graphs showing particle count (> 0.3 µm/0.1 L air) over time for the ultrasonic procedures in the single (i) and dual sensor (ii) setups. **A,** Standard high-volume suction (‘HVE’). **B,** Purevac® HVE Mirror Tip connected directly to the suction port (‘Purevac (−H)’). **C**, Purevac® HVE System which included the lightweight hose and adapter (‘Purevac (+H)’). **D**, Standard low-volume suction (‘LVE’). **E**, Ivory® ReLeaf™ hands-free suction device (‘ReLeaf’). **F**, DryShield® isolation system (‘DryShield’). **G**, No suction. Each line on the graphs represents one replicate. **H**, Bar charts showing normalised particle counts (mean ± SD, n = 5) for the ultrasonic procedures. Statistical analysis shown in Table 1

Consistently high levels of particles were detected during ultrasonic scaling and high-speed handpiece use in the absence of suction (Figs. 2G i,ii and 3E i,ii). In the absence of suction, the high-speed handpiece generated 3.4 times more particles/min than comparable time of use of the ultrasonic scaler when measured in the single sensor setup, and a comparable amount of aerosol when measured in the dual sensor setup.

**Fig. 3:**
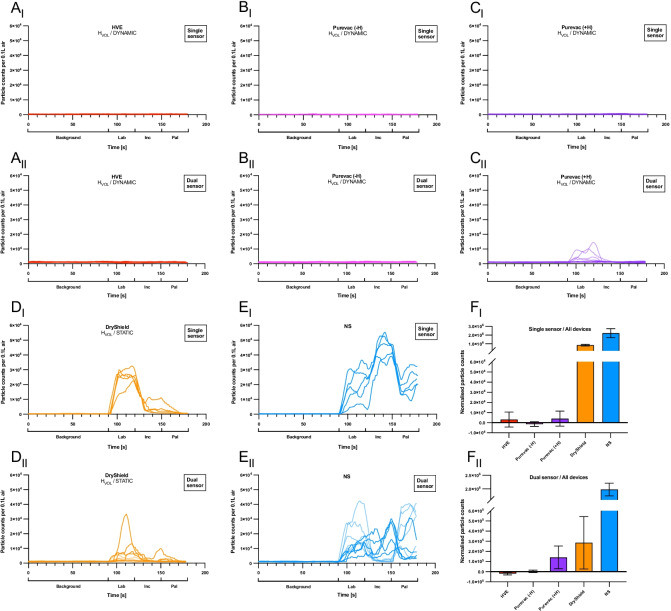
Particle counts for high-speed handpiece procedures**. A-E,** Line graphs showing particle count (> 0.3 µm/0.1 L air) over time for the high-speed handpiece procedures in the single **(i)** and dual sensor **(ii)** setups. **A**, Standard high-volume suction (‘HVE’). **B**, Purevac® HVE Mirror Tip connected directly to the suction port (‘Purevac (−H)’). **C**, Purevac® HVE System which included the lightweight hose and adapter (‘Purevac (+H)’). **D**, DryShield® Isolation System (‘DryShield’). **E**, No suction. Each line on the graphs represents one replicate. **F**, Bar charts showing normalised particle counts (mean ± SD, n = 5) for the ultrasonic procedures. Statistical analysis shown in Table 1

### High-volume dynamic suction devices reduce particle counts to background levels during ultrasonic scaling

Visual examination of the ultrasonic particle counts over time, in both the single and dual sensor setups, revealed the dynamic suction devices appear to keep the particle count at levels comparable to background, with only very infrequent particle ‘spikes’ observed (Fig. 2A i,ii – C i,ii). Conversely, the static suction devices show distinct spikes exceeding 10^4^ particles/0.1 L air during both a half and a full-mouth ultrasonic scale (Fig. 2D i,ii – F i,ii). These distinct spikes when using the static devices were observed at specific points during the procedures when working at the upper and lower labial areas of the mouth.

During ultrasonic scaling, both the ‘single sensor set up,’ and the ‘dual sensor set up’ revealed a statistically significant reduction in normalised particle counts with the use of any suction device, compared with no suction (*p* < 0.001) (Fig. 2H i,ii). In the single sensor set up, the use of dynamic suction devices gave a normalised particle count to near or below background levels (standard HVE: 2.3 × 10^3^ ± 2.1 × 10^4^; Purevac (−H): 4.5 × 10^3^ ± 1.2 × 10^4^; Purevac (+H): -2.8 × 10^3^ ± 1.3 × 10^4^). Using low-volume or static suction devices, the normalised particle counts were consistently above background (standard LVE: 4.0 × 10^4^ ± 2.6 × 10^4^; DryShield: 2.8 × 10^4^ ± 2.7 × 10^4^; ReLeaf: 3.8 × 10^4^ ± 2.6 × 10^4^) (Fig. 2H i). These results were replicated in the dual sensor setup, with dynamic suction devices giving normalised particle counts near to or below background levels, compared to the low-volume or static devices (Fig. 2H ii). All dynamic suction devices significantly outperformed standard LVE and ReLeaf with the dual sensor setup. With the single sensor setup, Purevac (+H) showed a statistically significant reduction in normalised particle counts compared with standard LVE (Table 1).

**Table 1:** Statistical comparisons of suction devices

Suction device comparisons	Single sensor setup	Dual sensor setup
**Ultrasonic scaling**		
HVE vs Purevac (−H)	–	–
HVE vs Purevac (+H)	–	–
HVE vs LVE	–	**
HVE vs ReLeaf	–	**
HVE vs DryShield	–	–
Purevac (−H) vs. Purevac (+H)	–	–
Purevac (−H) vs. LVE	–	***
Purevac (−H) vs. ReLeaf	–	***
Purevac (−H) vs. DryShield	–	–
Purevac (+H) vs. LVE	*	**
Purevac (+H) vs. ReLeaf	–	***
Purevac (+H) vs. DryShield	–	–
LVE vs. ReLeaf	–	–
LVE vs. DryShield	–	–
ReLeaf vs. DryShield	–	–
**High-speed handpiece**		
HVE vs. Purevac (−H)	–	–
HVE vs. Purevac (+H)	–	–
HVE vs. DryShield	***	*
Purevac (−H) vs. Purevac (+H)	–	–
Purevac (−H) vs. DryShield	***	*
Purevac (+H) vs. DryShield	***	–

Table showing a statistical comparison of the normalised particle counts in both the ultrasonic scaling and high-speed handpiece procedures, for all the suction devices: Standard high-volume suction (‘HVE’); Purevac® HVE Mirror Tip connected directly to the suction port (‘Purevac (−H)’); Purevac® HVE System which included the lightweight hose and adapter (‘Purevac (+H)’); Standard low volume suction (‘LVE’); Ivory® ReLeaf™ hands-free suction device (‘ReLeaf’); DryShield® Isolation System (‘DryShield’). Statistical comparison excludes no suction. All suction devices showed a statistically significant reduction in normalised particle count vs no suction (data not shown) (*p* < 0.001). Statistical analysis was carried out using one-way ANOVA with Tukey’s post-hoc test

* *p* < 0.05, ** *p* < 0.01, *** *p* < 0.001, '–' not significant

### High-volume dynamic suction devices reduce particle counts to background levels during high-speed handpiece use

Visual examination of the particle counts over time in both the dual and single sensor set ups in the high-speed handpiece arm of the study revealed comparable results to those observed using the ultrasonic scaler. Dynamic suction devices appeared to reduce the particle count to background levels (Fig. 3A i,ii – C i,ii); although there were small ‘spikes’ of aerosol escape detected when using Purevac (+ H) in the dual sensor setup (Fig. 3C ii) and numerically larger and more consistent spikes of aerosol escape when using DryShield in both the single and dual sensor setups (Fig. 3D i,ii). These distinct spikes were observed mainly during labial and incisal edge preparations.

Use of the high-speed handpiece with both single and dual sensor setups, revealed that all the suction devices tested resulted in a significant reduction in the normalised particle count compared with no suction (*p* < 0.001) (Fig. 3E i,ii). In the single sensor setup, the use of dynamic suction devices gave a normalised particle count close to or below background levels (standard HVE: 3.1 × 10^3^ ± 7.4 × 10^3^; Purevac (−H): − 1.4 × 10^3^ ± 2.2 × 10^3^; Purevac (+H) 4.0 × 10^3^ ± 7.4 × 10^3^), whereas the static DryShield device allowed escape of significantly more particles than all other dynamic suction devices (8.5 × 10^5^ ± 8.8 × 10^4^) (*p* < 0.001) (Fig. 3F i; Table 1). Similar results were observed in the dual sensor setup; use of all dynamic devices resulted in greater reduction in particle counts than DryShield (Fig. 3F ii; Table 1).

## Discussion

To the best of the authors’ knowledge this is the first study that presents a real-world and real-time clinical scenario using a variety of commonly used devices and conditions that would be frequently used by most dental professionals worldwide. Our results demonstrate that dynamic high-volume intra-oral suction devices that ‘follow’ the path of the particle generation at-source appear to effectively reduce the particle escape to near or below background levels for both ultrasonic scaling and high-speed handpiece procedures. These results are relevant as they quantify particle escape from the oral cavity, with and without the use of different intra-oral suction devices, measured comfortably within the working distance for a dental professional. This appears to be consistent with recent work carried out by Ehtezazi et al. [17] and Dudding et al. [18] who both present similar findings. Our results also indicate that static or low-volume suction devices allow notable periodic escape of particles and are less consistent in their reduction of particle escape during procedures, compared with dynamic high-volume suction devices. This again appears commensurate with the findings from Dudding et al. [18] with regards to the observed pattern of particle escape and effective mitigation with high-volume suction. Furthermore, several studies, including our own, have now demonstrated that high-speed handpieces generate greater particle numbers compared with ultrasonic scalers. [9, 10, 15, 16, 18, 19]

No single measures introduced to reduce the risk of SARS-CoV-2 transmission both in dental practice and in the wider community can ever be 100% effective. For example, a systematic review undertaken in 2020 showed the use of face masks amongst healthcare workers reduced the risk of respiratory virus infection by only 80% [25]. Reducing the direct risk from the patient themselves remains at the core of preventing the spread of SARS-CoV-2, such as through the use of telephone triage to ensure the absence of COVID-19 symptoms prior to an appointment [26]; however, this cannot simply be done based on the assessment of symptoms alone as robust evidence already exists of asymptomatic carriage of SARS-CoV-2 in patients who have attended dental appointments [27]. Studies have also shown coronaviruses may persist on surfaces—in some cases for a number of days [28]. The combination of asymptomatic carriage and virus persistence highlights the importance of maximising the prevention of aerosol escape at source, for every patient. Mitigation of aerosol particles by intra-oral suction is just one piece of a multi-factorial jigsaw of evidence that cannot be considered in isolation when considering the total risk of SARS-CoV-2 transmission in the dental setting; however, the evidence presented in our study suggests that the risk from AGPs themselves can be effectively managed.

The evaluation of aerosol escape in real-time using particle sensors allows an accurate and clear visual assessment of the effectiveness of each suction device during commonly performed AGPs; especially when considering the positioning and orientation of equipment which is constantly changing. There are, however, some limitations to this type of study. Firstly, our work was carried out using a dental simulation unit which clearly is not able to consider all patient derived factors such as saliva, coughing/breathing and difficulty of equipment access. However, it has previously been demonstrated that non-contaminated instruments are comparable with salivary-contaminated instruments with regards to the profile of particle distribution during ultrasonic scaling [18]. Secondly, accurately measuring any aerosol or particle distribution is challenging due to the unpredictable nature of the aerosol plume, both in terms of its volume and directionality. Our study was limited to three sensors positioned near to the oral cavity which may of course mean this setup fails to detect some of the particles escaping; however, there will always be compromise as for a meaningful simulation the detection systems have to allow sufficient access to the oral cavity. Thirdly, one single operator was used for both the ultrasonic scaling and high-speed handpiece procedures. It may be that inter-operator variability exists, which could be explored in further work. For the ultrasonic scaling, when using high-volume dynamic suction devices, the positioning of the suction and instrument was as per detailed manufacturer recommendations, however, this may not always be possible to replicate in all situations. Finally, our study used a ‘worst case scenario’ environment of zero air changes per hour, whereas many previous studies have utilised consistent mechanical ventilation when measuring aerosols [5, 9–15, 17]; although it should be noted that these studies have largely focused on the concept of fallow-time, compared with our approach of measuring particle-escape directly from the oral cavity. Of note, whilst we have used ‘particles’ to encompass all secretions detected by the sensors, the diverse terminology surrounding the subject of AGPs—including particles, aerosols, splatter and fallow-time – is likely to be a confounder in the literature.

## Conclusion

Our results demonstrate the effective mitigation of aerosols generated from ultrasonic scaling and high-speed handpiece procedures using high-volume dynamic intra-oral suction. We have shown that aerosol particle generation varies between different aerosol-generating devices and can be mitigated at varying levels depending on the choice of intra-oral suction, potentially to background levels. Further work is required to explore several factors, for example potential variations between operators and/or additional sensor positions. However, with the uncertainty surrounding the risk of SARS-CoV-2 transmission in dental practices and, in some cases, the restrictions still imposed on dental care professionals, we hope the data presented here offer reasonable suggestions towards the choice of suction equipment and its use as a logical and simple starting point to help inform future decisions on AGPs and their mitigation.

## Availability of data and materials:

The datasets used and analysed during this study are available from Shauna Culshaw on reasonable request.

## Supplementary Information

**Additional file 1**. Study setup within the Clinical Research Facility. Plan and section view of the study setup within the Clinical Research Facility; and figures showing the positioning of the particle sensors relative to the incisal edges, for both the single and dual sensor setups (drawings created by Dr Piotr J. Lesniak).

**Additional file 2**. Creating files for data storage and Bash code for processing of the raw particle sensor data.

**Additional file 3**. Aerosol generating devices and intra-oral suction devices. Aerosol generating devices and intra-oral suction devices. **A**, Cavitron® Powerline® 1000 30K Ultrasonic Insert. **B**, Midwest Stylus® Plus Handpiece. **C**, Standard high-volume suction. **D**, Purevac® HVE Mirror Tip. **E**, DryShield® Isolation System. **F**, Ivory® ReLeaf™ hands-free suction device. **G**, Standard low-volume suction. **H**, Aerosol generation and mitigation.

**Additional file 4**. Aerosol generation in the absence of suction. Aerosol generation in the absence of suction, here showing the use of a high-speed handpiece in the dual sensor setup.

## Abbreviations

AGP: Aerosol generating procedure
COVID-19: Coronavirus disease 2019
SARS-CoV-2: Severe acute respiratory syndrome coronavirus 2
HVE: High-volume evacuation
LVE: Low-volume evacuation
AUC: Area under curve
