# Supplementary figures and images for: Aerosol reduction efficacy of different intra-oral suction devices during ultrasonic scaling and high-speed handpiece use
Source: BMC Oral Health. 2022 Sep 6;22:388. doi: 10.1186/s12903-022-02386-w (PMC9447970; doi:10.1186/s12903-022-02386-w)

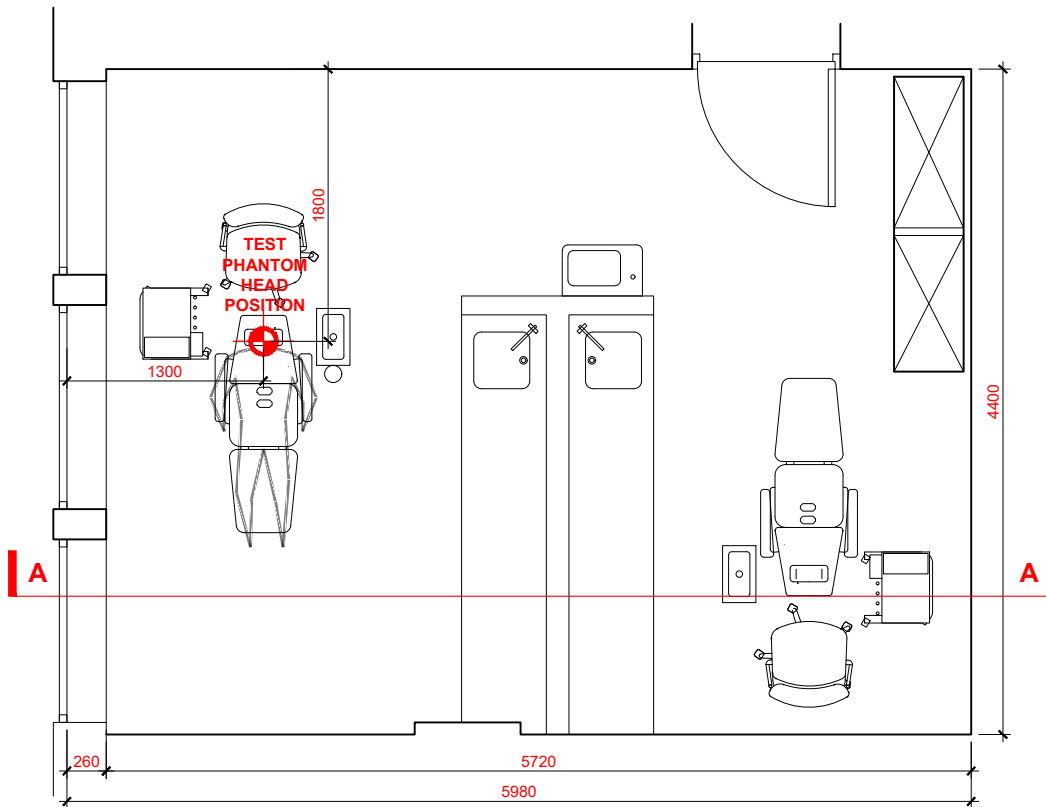

**PLAN VIEW**

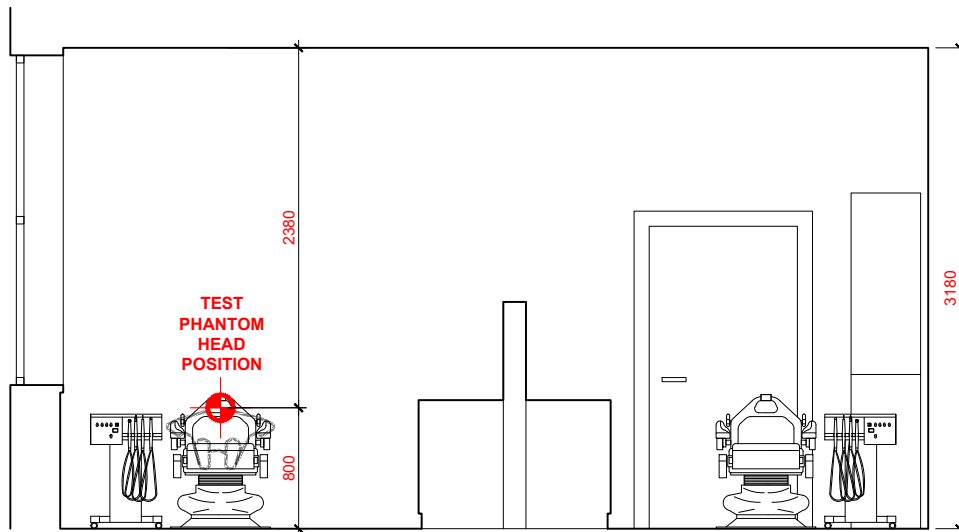

**SECTION A-A**

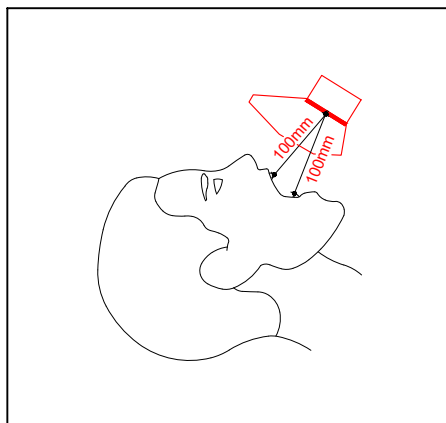

**SINGLE SENSOR SETUP**

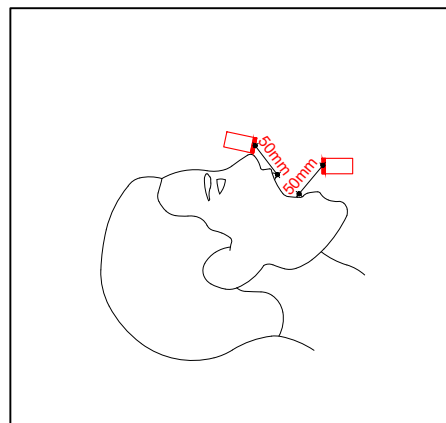

**DUAL SENSOR SETUP**

Supplement: Supplementary file 1 — Additional file 1. Study setup within the Clinical Research Facility. Plan and section view of the study setup within the Clinical Research Facility; and figures showing the positioning of the particle sensors relative to the incisal edges, for both the single and dual sensor setups (drawings created by Dr Piotr J. Lesniak). [file 12903_2022_2386_MOESM1_ESM.pdf]

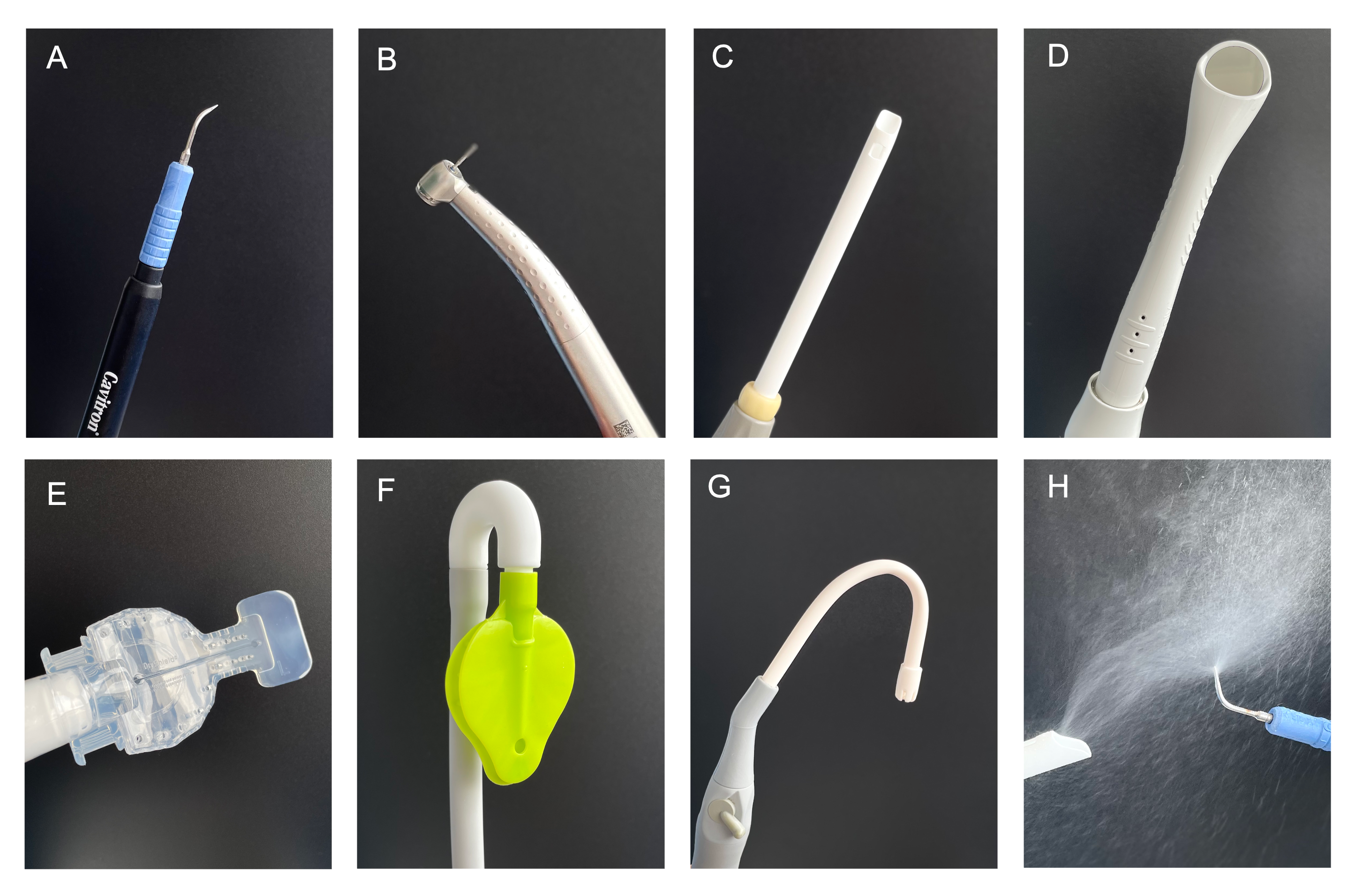

Supplement: Supplementary file 3 — Additional file 3. Aerosol generating devices and intra-oral suction devices. Aerosol generating devices and intra-oral suction devices. A, Cavitron® Powerline® 1000 30K Ultrasonic Insert. B, Midwest Stylus® Plus Handpiece. C, Standard high-volume suction. D, Purevac® HVE Mirror Tip. E, DryShield® Isolation System. F, Ivory® ReLeaf™ hands-free suction device. G, Standard low-volume suction. H, Aerosol generation and mitigation. [file 12903_2022_2386_MOESM3_ESM.png]

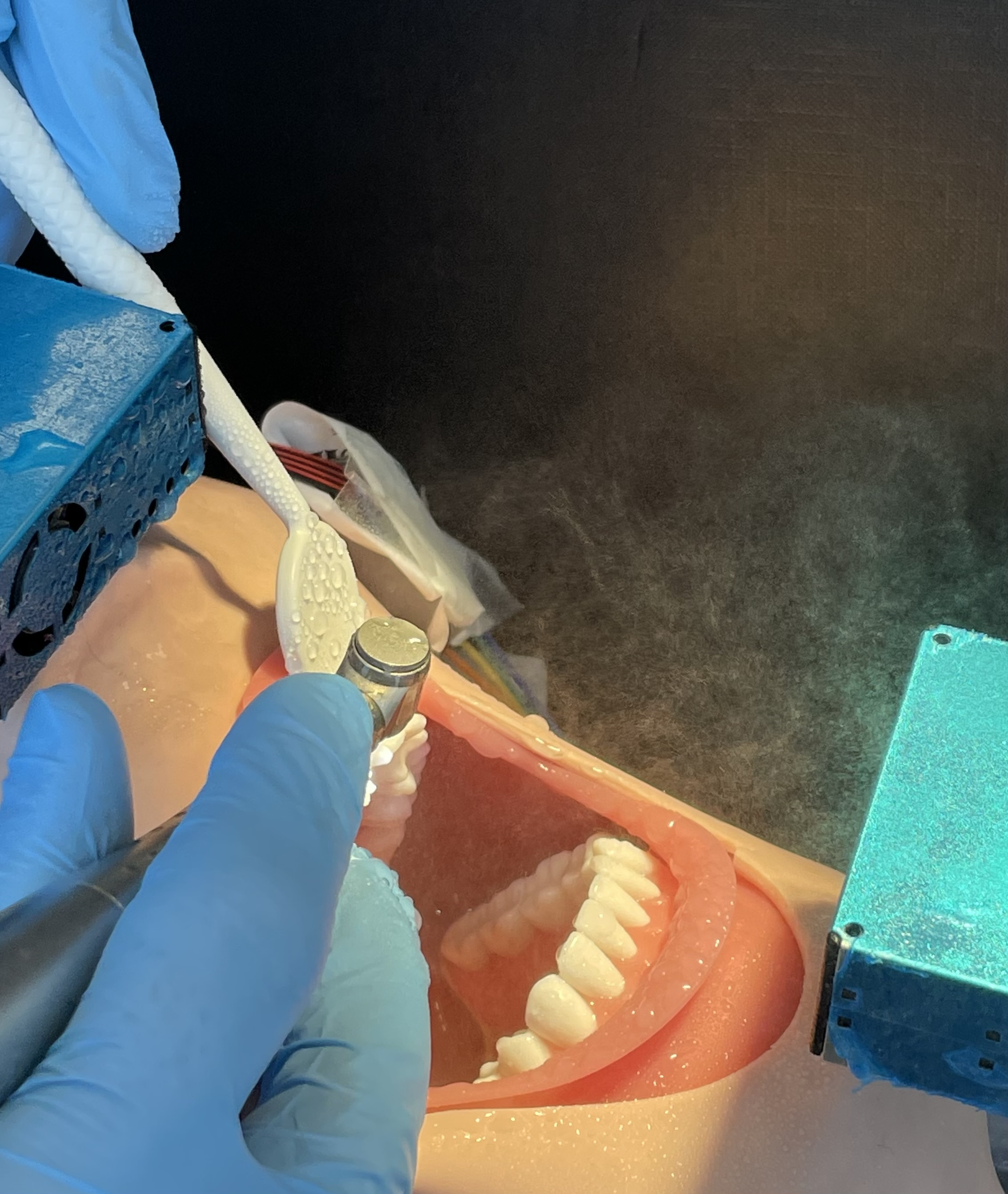

Supplement: Supplementary file 4 — Additional file 4. Aerosol generation in the absence of suction. Aerosol generation in the absence of suction, here showing the use of a high-speed handpiece in the dual sensor setup. [file 12903_2022_2386_MOESM4_ESM.png]
