# Supplementary material for: Aerosol reduction efficacy of different intra-oral suction devices during ultrasonic scaling and high-speed handpiece use
Source: BMC Oral Health. 2022 Sep 6;22:388. doi: 10.1186/s12903-022-02386-w (PMC9447970; doi:10.1186/s12903-022-02386-w)
Supplement: Supplementary file 2 — Additional file 2. Creating files for data storage and Bash code for processing of the raw particle sensor data. [file 12903_2022_2386_MOESM2_ESM.docx]

**SUPPLEMENTARY TEXT 1. Creating files for raw data storage and Bash code for data processing.**

1. Make a list of test names separated by a single space, for example:

| DeviceA_R1 DeviceB_R1 DeviceC_R1 |
| --- |

1. Implement the list into the following code:

| for i in DeviceA_R1 DeviceB_R1 DeviceC_R1; do  touch "${i}.txt"  touch "${i}_RAW.txt"  echo "grep 'YYYY-MM-DD' ${i}.txt \| awk {'print \$2'}\| paste - >${i}_time.txt >${i}_time.txt ; grep '>' ${i}.txt \| awk {'print \$5'}\| paste - - - - - - >${i}_particles.txt && paste ${i}_time.txt ${i}_particles.txt \| column -s $'\t' -t > ~/${i}_export.txt" >"${i}_code.txt"  done |
| --- |

***Please note:*** date 'YYYY-MM-DD' refers to the date set up on the RaspberryPi sensor.

1. Launching the code in Terminal generates device-specific text files in the Home folder (Shift+cmd+H):

- ‘DeviceA_R1_RAW.txt’, ‘DeviceB_R1_RAW.txt, ‘DeviceC_R1_RAW.txt - empty device-specific files for saving the raw data,
- ‘DeviceA_R1.txt’, ‘DeviceB_R1.txt, ‘DeviceC_R1.txt - empty device-specific files for saving the readings only,
- ‘DeviceA_R1_code.txt’, ‘DeviceB_R1_code.txt’, ‘DeviceC_R1_code.txt’ - files containing device-specific Bash code to be used in the Terminal to export time and particle counts readings from the raw data files ‘DeviceA_R1.txt’, ‘DeviceB_R1.txt’, ‘DeviceC_R1.txt’.

**RaspberryPi setup for Macbook:**

1. Connect MacBook to the RaspberryPi2 and Wi-Fi dongle.
2. Launch a program (e.g., LanScan) that will scan the networks in search for the RaspberryPi2 IP address which should have a XXX.XXX.X.XXX format.
3. Launch Terminal.
4. Terminal commands for starting the sensor:

| ssh pi@XXX.XXX.X.XXX  password: (invisible)  cd Pimoroni  cd enviroplus  cd examples  ./particulates.py |
| --- |

1. Readings should appear on the screen.
2. Stop the run (ctr+C).
3. Copy the full text into the raw text file (e.g., DeviceA_R1_RAW.txt), and save.
4. Select the readings only and copy (cmd+C) them into the empty text file (e.g., DeviceA_R1.txt), then save (cmd+S).
5. Close both files (cmd+Q).

*Please note:* always use *.txt and not *.rtf file format – it is best to change the default settings in the TextEdit application, or alternatively, text file can be converted to Unicode (UTF-8) by pressing shift+cmd+T.

Raw data processing:

1. Open the corresponding text file with the device-specific code (e.g., DeviceA_R1_code.txt) and run it in the Terminal.

| grep 'YYYY-MM-DD' DeviceA_R1.txt \| awk {'print $2'}\| paste - >DeviceA_R1_time.txt >DeviceA_R1_time.txt ; grep '>' DeviceA_R1.txt \| awk {'print $5'}\| paste - - - - - - >DeviceA_R1_particles.txt && paste DeviceA_R1_time.txt DeviceA_R1_particles.txt \| column -s $' ' -t > ~/DeviceA_R1_export.txt |
| --- |

1. Following files should appear in the Home folder:

- DeviceA_R1_time.txt
- DeviceA_R1_particles.txt
- DeviceA_R1_export.txt

1. Open a blank Excel file and import the *_export.txt file (e.g. DeviceA_R1_export.txt) by choosing Data/Get Data/Choose the file/Get Data/Next/Important - set the field widths/Next/Finish/OK and save (cmd+S) as e.g. (e.g. DeviceA_R1_imported.xlsx), then close the file (cmd+Q).

*Please note:* When setting the field widths the whole dataset needs to be controlled, otherwise the particle readings may be divided and placed into the wrong columns.
